# Semantic Network Analysis Reveals Opposing Online Representations of the Search Term “GMO”

**DOI:** 10.1002/gch2.201700082

**Published:** 2017-12-27

**Authors:** Ke Jiang, Brittany N. Anderton, Pamela C. Ronald, George A. Barnett

**Affiliations:** ^1^ Manship School of Mass Communication Louisiana State University 211 Journalism Building Baton Rouge LA 70803 USA; ^2^ Department of Neurobiology Physiology and Behavior University of California, Davis 1 Shields Avenue Davis CA 95616 USA; ^3^ Department of Plant Pathology and the Genome Center University of California, Davis 1 Shields Avenue Davis CA 95616 USA; ^4^ Department of Communication University of California, Davis 1 Shields Avenue Davis CA 95616 USA

**Keywords:** concept co‐occurrence, genetically engineered foods, GMO, online information, semantic network analysis

## Abstract

Making sound food and agriculture decisions is important for global society and the environment. Experts tend to view crop genetic engineering, a technology that can improve yields and minimize impacts on the environment, more favorably than the public. Because there is a causal relationship between public opinion and public policy, it is important to understand how opinions about genetically engineered (GE) crops are influenced. The public increasingly seeks science information on the Internet. Here, semantic network analysis is performed to characterize the presentation of the term “GMO (genetically modified organism),” a proxy for food developed from GE crops, on the web. Texts from three sources are analyzed: U.S. federal websites, top pages from a Google search, and online news titles. We found that the framing and sentiment (positive, neutral, or negative attitudes) of “GMO” varies across these sources. It is described how differences in the portrayal of GE food by each source might affect public opinion. A current understanding of the types of information individuals may encounter online can provide insight into public opinion toward GE food. In turn, this knowledge can guide teaching and communication efforts by the scientific community to promote informed decision‐making about agricultural biotechnologies.

## 1 Introduction

Sensible decision‐making about food and agriculture is a top public policy challenge worldwide. Genetically engineered (GE) crops were introduced to American markets over 20 years ago. These crops have numerous potential benefits, including increased yield and decreased environmental impact. A growing body of evidence indicates that food derived from GE crops (hereafter referred to as GE food) is as safe to eat as food derived from conventionally grown crops.^1^ Despite these conclusions, there remains a large gap between public opinion and scientific consensus on the safety of GE food.^1^, ^2^, ^3^, ^4^ Because public opinion is likely to influence policy decisions about GE food,^5^ it is important to understand how opinions are influenced.

Framing is the process of tailoring messages so that they “resonate with [the] core values and assumptions of others.”^6^ Framing of science and health‐related subjects in mass media can influence public opinion.^7^, ^8^ For example, it is one method by which actors—individuals or groups who seek to “mobilize” the public in order to change or maintain a policy decision—attract or drive attention away from an issue.^5^ Prior research suggests that individuals' perceptions of media reporting about GE foods align with their perceptions of GE food risks and benefits.^9^ Therefore, how GE food—and other socioscientific issues such as nuclear power—10 is portrayed in mass media likely influences public opinion and behavior.

Several recent studies have evaluated the framing of the GE food debate in mass media.^11^, ^12^, ^13^, ^14^ Expanding upon these findings, Tosun and Schaub characterized the strategies used by opposing groups to mobilize the European public for or against GE crops.^5^ However, these studies focused on GE coverage solely in newspapers or online news. We sought to characterize the presentation of GE crops and food on the Internet, a source that is increasingly relied on by consumers for information about science.^15^, ^16^ Because the Internet is not homogeneous, it is important to consider the possible distinct venues trafficked by nonexperts who seek scientific information on the web. We reasoned that three sources likely predominate for those seeking information about GE food: Google searches, online news, and regulatory websites. To our knowledge, this is the first study to simultaneously compare framing by multiple online sources for a single scientific issue.

**Figure 1:**
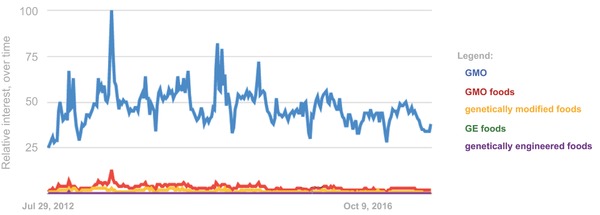
Worldwide search interest results for five search terms related to genetically engineered food and crops from the past five years. The *y*‐axis represents search interest relative to the highest point on the chart for the given length of time (Source: Google Trends).

We predicted that the framing of GE food would vary with each source. We used the semantic network analysis (SMA), a form of content analysis that identifies the network of associations between concepts expressed in text, to compare the image of “GMO” (genetically modified organism)–a proxy for GE food—portrayed by each source. Although the term is not useful in a scientific or agricultural context because it is ill‐defined,^17^ we chose “GMO” because it is the most‐searched term related to food biotechnology (**Figure** **1**).^18^ Our analysis indicates that there is minimal overlap in the semantic networks derived from the three sources. Further, we provide empirical evidence that different online sources portray contrasting sentiments on a controversial science subject. These results provide insight into how the Internet can influence public opinion about GE food. In turn, this knowledge can guide teaching and communication efforts by the scientific community to promote informed decision‐making about agricultural biotechnologies.

## 2 Research Methods

Computer‐assisted SMA is a form of content analysis that identifies the network of associations between concepts expressed in a text.^19^, ^20^ This approach has been previously applied to assess media coverage of the human papillomavirus (HPV) vaccine,^21^, ^22^ another controversial science subject. Rooted in the cognitive paradigm,^23^ and the tradition of frame semantics in linguistics,^24^ scholars have argued that words are hierarchically clustered in memory.^25^ Thus, spatial models (e.g., networks) that illustrate the relations among words are representative of meaning.^26^ The structured representations of the connections between concepts or terms are regarded as semantic networks.^27^ Sentiment analysis, the process of identifying and categorizing opinions expressed in text to determine whether the stated attitude toward a particular subject is positive, negative, or neutral,^28^ is often coupled to SMA.

This study used network analysis software including ConText,^29^ Gephi,^30^ and UCINET^31^ to analyze and visualize the content of texts containing the term “GMO” from United States federal and regulatory websites including the Food and Drug Administration, National Institutes of Health, National Science Foundation, US Department of Agriculture, Environmental Protection Agency, National Academies of Science, Engineering, and Medicine, and American Association for the Advancement of Science, which were retrieved on August 12, 2016; ten websites listed on the first page of the results of searching “GMO” on Google, retrieved on June 2, 2016; and 660 titles from a Google News search of “GMO” for one year, May 2015 to May 2016. Ten websites from a Google search were examined because the majority of Internet users (54.1%) view only one page of search results and 97.7% view less than ten pages of results.^32^ In order to avoid search engine optimization, the Google searches were performed in Private Browsing mode in the Safari web browser. The time frame for the news title analysis was chosen to insure that the sample would be sufficiently robust for the text analysis. The inquiry was limited to the titles of news articles so that a manageable amount of semantic information would be available for analysis. Three semantic networks were created based on the analysis of word co‐occurrence: one for the articles from the federal websites (FW), another for the Google search top pages (GP), and a third based on Google online news titles (ON).

This study measures the prominence of concepts related to “GMO” through the analysis of word centrality, which reflects the location and importance of a word in relation to other words in a network.^33^, ^34^ It also examines concept associations by characterizing the subclusters that comprise the semantic networks, along with the frequency with which concepts co‐occur. The main research questions were as follows:

R1: What are the most central words in each network?
R2: How does framing of “GMO” compare and contrast between networks?
R3: Is the framing of “GMO” largely positive, negative, or neutral in each source?

The first step in the research, after collecting the raw data (texts) from the online sources, was to edit the texts. Syntactically functional words (e.g., a, an, the) were removed; different forms of the same word (e.g., modify and modified) were stemmed. We chose not to collapse the terms “GMO'” and “GM,” because although they have similar meaning, the former describes an organism (or product) while the latter describes a process. Then, the frequency of the words in the three datasets was calculated. The words whose frequency was equal to or greater than the mean frequency of each dataset were selected for analysis. The mean frequency of occurrence was 5, 4, and 3 in the texts retrieved from federal websites, Google search top pages, and titles of Google online news, respectively.

The second step was to generate semantic matrices from the edited texts. Links between words in the semantic networks were based on word co‐occurrence. Miller^35^ argued that people's working memory has a capacity of “seven plus‐or‐minus two” chunks, indicating people can process seven meaningful units, plus or minus two, at a time. Based on this argument, words that occurred within seven words of each other in the edited texts were considered connected.^36^ The first two steps were conducted using the ConText software.

In the third step, the three semantic networks were examined using UCINET and Gephi, which are software developed for network analysis, graphics, and statistical computing. UCINET calculates the normalized degree and eigenvector centralities of each word in the three semantic networks. Eigenvector centrality is a measure of a word's overall location and importance in relation to other words in a network.^37^ For example, a word's eigenvector centrality increases if it is linked to more central words. Gephi calculates the subclusters within networks by conducting modularity analysis^38^ and creates visual maps of networks. Co‐occurrence of words in the same subgroup reflects a high frequency of co‐occurrence in the text. The subgroups are differentially colored for illustrative purposes.

The sentiment analysis algorithm from ConText was applied to determine if the three representations of “GMO” may be characterized as positive, negative, or neutral.^29^ The software uses the MPQA subjectivity lexicon (http://mpqa.cs.pitt.edu/lexicons/subj_lexicon/). The lexicon consists of three types of polarity weights: positive, negative, and neutral. For this analysis, the input text is first stemmed; all variants of a word using different parts of speech are combined. Then, if a term coincides with a lexicon entry, it is tagged with the given polarity in the lexicon.^39^ The sentiment of each applicable word is indicated in **Table** **1**.

**Table 1:** Summary output of semantic network analysis (SMA). The top 50 words with greatest eigenvector centralities are shown for texts related to “GMO” derived from federal websites, Google top pages, and Google online news titles. (Eigenvector centrality has been normalized. Common words in the three semantic networks are highlighted in italics; unique words in each semantic network are highlighted in bold. * indicates the words are positive. # indicates the words are negative)

	Federal websites		Google first page		Online news titles	
	Word	Eigen	Word	Eigen	Word	Eigen
1	*genetic*	**0.211**	*food*	*0.198*	label	0.336
2	biotech	0.207	*genetic*	*0.198*	*food*	*0.279*
3	plant	0.207	*crop*	*0.198*	*crop*	*0.252*
4	*food*	*0.206*	GM	0.193	*genetic*	*0.233*
5	*crop*	*0.194*	**study**	**0.178**	*modify*	*0.199*
6	production	0.194	engineering	0.177	**ban**	**0.186**
7	**regulation**	**0.191**	*modify*	*0.177*	**Monsanto**	**0.180**
8	* safe	*0.187*	plant	0.175	* approval	**0.170**
9	**EPA**	**0.184**	production	0.164	farmer	0.147
10	environment	0.183	produce	0.163	**law**	**0.141**
11	**engineer**	**0.181**	* safe	*0.162*	**# fight**	**0.135**
12	produce	0.173	development	0.157	**debate**	**0.133**
13	development	0.173	health	0.156	**mosquito**	**0.132**
14	**agency**	**0.171**	technology	0.155	**stop**	**0.123**
15	pesticide	0.170	make	0.154	**court**	**0.122**
16	animal	0.169	animal	0.154	**China**	**0.119**
17	USDA	0.168	**rice**	**0.152**	study	0.117
18	* protection	**0.161**	**gene**	**0.152**	**nation**	**0.117**
19	agriculture	0.160	human	0.152	FDA	0.115
20	organism	0.157	organism	0.149	**# challenge**	**0.110**
21	FDA	0.157	**scientist**	**0.147**	**import**	**0.110**
22	health	0.154	**Bt**	**0.147**	**company**	**0.109**
23	**information**	**0.148**	**people**	**0.146**	**# trial**	**0.109**
24	**requirement**	**0.147**	**world**	**0.146**	**join**	**0.108**
25	**process**	**0.147**	# risk	0.144	USDA	0.108
26	engineering	0.146	research	0.143	**call**	**0.108**
27	human	0.143	GE	0.142	**bill**	**0.107**
28	# risk	0.143	agriculture	0.142	**move**	**0.107**
29	**# pest**	**0.142**	**protein**	**0.141**	**Vermont**	**0.106**
30	* ensure	**0.141**	environment	0.141	**industry**	**0.106**
31	**variety**	**0.139**	**corn**	**0.138**	* safe	*0.105*
32	test	0.135	**growth**	**0.138**	**organic**	**0.104**
33	**evaluate**	**0.134**	**eat**	**0.135**	**legislature**	**0.104**
34	**provide**	**0.134**	**papaya**	**0.133**	**big**	**0.103**
35	make	0.133	farmer	0.132	**# illegal**	**0.101**
36	GE	0.131	feed	0.130	**Senate**	**0.100**
37	science	0.130	**greenpeace**	**0.130**	**end**	**0.096**
38	**insect**	**0.128**	test	0.129	**Florida**	**0.096**
39	**derive**	**0.128**	**result**	**0.126**	**mandatory**	**0.095**
40	**# resistance**	**0.127**	**nature**	**0.126**	**Zika**	**0.095**
41	**marketing**	**0.127**	pesticide	0.125	**lawsuit**	**0.093**
42	*modify*	*0.126*	science	0.125	**# kill**	**0.092**
43	**review**	**0.125**	**toxin**	**0.124**	**deal**	**0.091**
44	**trait**	**0.125**	**golden**	**0.123**	**Philippines**	**0.091**
45	research	0.124	**# virus**	**0.122**	**county**	**0.090**
46	**issue**	**0.123**	**effect**	**0.119**	**plan**	**0.090**
47	**assessment**	**0.122**	**transgenic**	**0.118**	**battle**	**0.090**
48	feed	0.117	**# problem**	**0.117**	**campaign**	**0.088**
49	**USA**	**0.117**	label	0.116	**soybean**	**0.088**
50	technology	0.117	biotech	0.116	GM	0.088

## 3 Results

Table 1 lists the 50 words with greatest normalized eigenvector centralities for FW, GP, and ON. There are only five common words across the three semantic networks: crop, food, genetic, modify, and safe. Specifically, crop, food, and genetic have the greatest eigenvector centralities in all three semantic networks. While safe is the eighth most central word in FW, its centralities rank 11 and 31 in GP and ON, respectively. Regulation, EPA, engineer, agency, and protection are the unique words with greatest eigenvector centralities in FW. Study, rice, gene, scientist, and Bt are the unique words with greatest eigenvector centralities in GP. Ban, Monsanto, approval, law, and fight are the unique and central words in ON.

**Figure 2:**
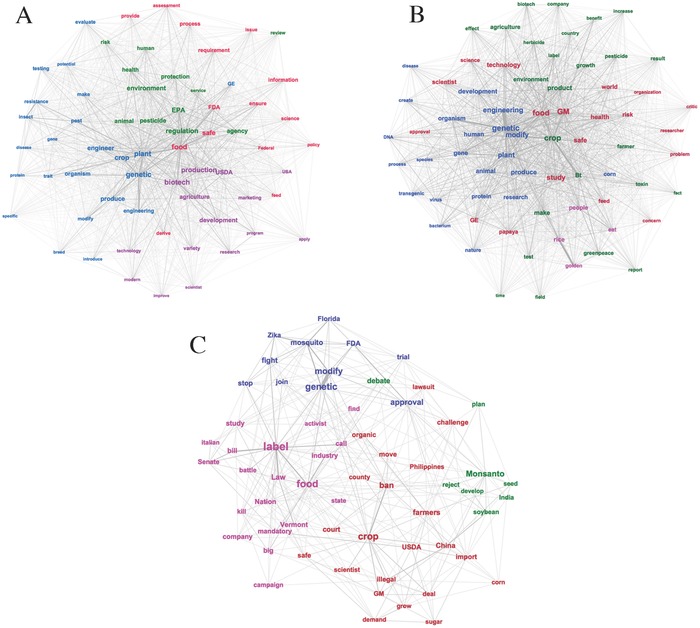
Semantic networks representing “GMO” online. A) Semantic network of federal websites. B) Semantic network of Google search top pages. C) Semantic network of online news titles. Note: Summary of colored clusters derived by modularity analysis in Table 2.

**Figure** **2**A–C presents graphical representations of FW, GP, and ON's semantic networks. The minimum strength for displaying a word link in FW, GP, and ON are the mean number of links in each network (e.g., FW = 63, GP = 74, ON = 12). The size of the label indicates the words' normalized eigenvector centrality. For Figure 2B,C (GP and ON, respectively), the most central word, GMO, was removed from the network because its high centrality linked all the other concepts together into a single group and distorted the results. GMO also does not appear in Figure 2A (FW), because its eigenvector centrality fell below 63, the mean number of links in FW, and therefore did not meet the cutoff for inclusion in that network.

Modularity analysis identified four word clusters in each semantic network (colored groups in Figure 2A–C). **Table** **2** summarizes the subclusters, including overall theme, percent share of the network, and the five strongest word associations in each cluster. The theme of the most prominent cluster in FW was genetic engineering of crops and associated traits (blue cluster; 32% share of network). This cluster centers about the term genetic, which is closely linked to engineering, plant, and crop. Lesser clusters in FW are centered about the themes of environmental safety and regulation (green cluster; 20.57% share of network); food safety and regulation (red cluster; 22.86% share of network); and biotechnology research and development (purple cluster; 24.57% share of network). Specifically, the most central word in the green cluster is regulation, which has strong associations with EPA, pesticide, and agency. The most central terms in the red cluster were food and safe, which are strongly associated with each other. Also, safe in the red cluster has strong associations with ensure and FDA. The most central word in the purple cluster is biotech. It is strongly linked with agriculture, production, development, and research.

**Table 2:** Summary output of cluster analysis. The themes, top word associations, and percent share of respective network are shown for sub clusters in federal websites, online news titles, and Google top pages. The clusters are represented as red, blue, green, and purple in Figure 2; similar colors across networks do not necessarily indicate a link between subclusters

		Theme		Top associations				Association count		Cluster color		Share of network [%]
Federal websites		Food safety and regulation		food		safe		39		Red		22.86
				food		FDA		39				
				food		drug		19				
				ensure		safe		16				
				FDA		safe		14				
		GE crops and traits		genetic		engineering		46		Blue		32
				genetic		plant		34				
				insect		resistance		30				
				genetic		crop		28				
				engineer		plant		26				
		Environmental safety and regulation		regulation		EPA		36		Green		20.57
				EPA		pesticide		29				
				regulation		pesticide		24				
				regulation		agency		18				
				environment		health		18				
		Biotechnology research & development		biotech		agriculture		33		Purple		24.57
				biotech		production		22				
				agriculture		USDA		16				
				biotech		development		15				
				biotech		research		14				
Google top pages		GE food safety		GM		food		34		Red		29.51
				GM		technology		14				
				food		safe		14				
				GM		safe		11				
				study		safe		11				
		GE in plants		genetic		modify		154		Blue		24.59
				genetic		engineering		90				
				genetic		organism		39				
				genetic		plant		26				
				gene		plant		22				
		GE crops and traits		crop		Bt		43		Green		25.68
				crop		environment		15				
				Bt		greenpeace		12				
				crop		herbicide		10				
				crop		benefit		10				
		Biofortified golden rice		rice		golden		50		Purple		20.22
				beta		carotene		35				
				rice		beta		17				
				rice		carotene		16				
				vitamin		deficiency		14				
Online news titles		Global trade of GE crops		crop		ban		7		Red		36.07
				crop		GM		4				
				China		illegal		4				
				crop		illegal		3				
				ban		import		2				
		Genetically engineered mosquitoes (Zika)		genetic		modify		23		Blue		18.03
				mosquito		modify		7				
				genetic		mosquito		7				
				mosquito		Zika		7				
				mosquito		Florida		6				
		Agrichemical industry – research and trade		Monsanto		Bayer		4		Green		13.11
				Monsanto		cotton		3				
				trade		reject		3				
				field		dilemma		3				
				soybean		dilemma		3				
		GE food labeling – legislation		label		law		9		Purple		32.79
				label		bill		8				
				label		food		7				
				Vermont		law		4				
				Senate		bill		4				

The four word clusters in GP had comparable shares of the overall network, ranging from 20.22% to 29.51%. The theme of the top cluster (red, 29.51% share of network) is GE food safety; the themes of the two middle clusters (blue, 24.59% share of network; green, 25.68% share of network) are GE in plants and GE crops and traits, respectively; and the theme of the least prominent cluster (purple, 20.22% share of network) is biofortified Golden Rice. Specifically, the red cluster is centered about GM, food, and study, in which GM has the strongest association with food, and study has the strongest association with safe. The blue cluster is centered about genetic, which is closely linked to modify and engineering. The green cluster is centered about crop, which is closely linked to Bt, environment, herbicide, and benefit. The purple cluster has the fewest words and is centered about rice, which is closely associated with golden, beta, and carotene.

We identified two similarly prominent clusters in ON. One centers about the theme of global trade of GE crops (red cluster; 36.07% share of network). The most central word in this cluster is crop, which has close associations with ban, GM, and illegal. The theme of the other prominent cluster is GE food labeling and associated legislation (purple cluster; 32.79% share of network). Its most central word is label, which is strongly associated with law, bill, and food. The two lesser clusters in ON center are about the themes of trade and testing in the agrichemical industry (green cluster; 13.11% share of network) and genetically engineered mosquitoes (blue cluster; 18.03% share of network). The green cluster has the least number of words in ON, and its most central word is Monsanto, which is closely associated with Bayer and cotton. Genetic is the most central word in the blue cluster, and has the strongest association with modify and mosquito.

The blue clusters are most similar across the three semantic networks, with genetic being the most central word in each. It has strongest associations with engineering and plant in FW, modify and engineering in GP, and modify and mosquito in ON.

The results of the sentiment analysis indicate that for the words with greatest eigenvector centralities, FW has the highest number of positive words (3), GP has the least number of words with either positive or negative sentiments, and ON has the most negative words (5) (see Table 1).

## 4 Discussion

Media discourse, particularly framing in mass media, can influence public opinion about science‐related subjects. It is likely that where individuals seek their science information has contrasting effects on their attitudes, due to differences in content. There remains a large gap between public opinion and scientific consensus on the safety of food derived from GE crops. We characterized how “GMO,” the most highly searched term related to GE food in Google (Figure 1), is presented in different areas of the web. We sought to determine whether different sources provide varied sentiments on GE food. Our findings indicate that presentation of the term “GMO” differs among three sources on the Internet: Federal webpages, top Google search pages, and online news titles.

Only 10% of the most central words were shared by all three sources, while a much larger proportion (between 42–78%) of words were unique to each source. This indicates that information about food derived from GE crops is portrayed differently by federal websites, highly trafficked websites, and online news. For example, we found that online news titles were unique in their use of terms suggestive of argumentation, including ban, fight, debate, challenge, kill, and battle (Table 2). Similar results have been identified in analyses of agricultural biotechnology and/or GMO coverage in newspapers from the Philippines, United States, and United Kingdom, which characterized coverage as containing drama, controversy, and debate between potential risks versus potential benefits.^12^, ^14^ This focus on argumentation and controversy may impart a lack of confidence in the safety or usefulness of commercially available GE products. Alternatively, federal websites' unique use of words related to the regulatory process, including regulation, protection, ensure, evaluate, review, and assessment (Table 2), may invoke trust in the safety of commercial GE crops. We observed a technical frame in top pages from a Google search of “GMO”; the unique words in GP largely related to common GE crop topics or applications, such as Bt, protein, corn, papaya/virus (likely in reference to papaya ringspot virus), and rice/golden (likely in reference to biofortified golden rice). This indicates that highly trafficked websites about food derived from GE crops serve to inform the public about relevant GE applications. It is unclear whether this information is expected to have a positive or negative impact on public opinion.

We also found that the sources varied in the sentiment of their most central words. For example, online news titles and Google search pages had more negative than positive terms, while federal websites had an equal number of negative and positive terms (Table 2). Thus, beyond differences in content portrayed by each source, these sources also provide information that is framed with varied sentiment. Because individuals' perceptions of media reporting about GE foods has been shown to align with their perceptions of GE food risks and benefits,^9^ it is likely that negative portrayals of GE food on the Internet impart negative attitudes toward this technology.

Prior research suggests that contrasting worldviews produce different risk discourses regarding GMOs.^13^ Therefore, the diverse portrayals of GE food we identified in three sources on the worldwide web likely represent the varied—and perhaps opposing—worldviews of the sources' predominant authors. For example, the authors of GMO‐related texts on regulatory websites may view genetic engineering as a tool to enhance agricultural practices, while journalists may view the same topic as threatening to human health or the environment. Additionally, prior work has found that opposing portrayals of GE food in the media serve to promote attitudes and behavior for or against the technology.^5^ From this, we predict that online news generally mobilizes the public against, while federal or government websites generally mobilize the public for, GE food. The effect of top Google search pages is less clear, as a cohesive frame was not determined. Further research is needed to empirically determine whether these predictions hold true.

## 5 Conclusion

Media discourse can influence public opinion on a range of socioscientific issues. Here, we show that three online sources provide information that is framed differently for the same topic, “GMO,” a proxy for GE food. One limitation of this study is that we did not evaluate the validity of information provided by each source – that is, whether or not their information is scientifically accurate. We simply characterized the display of information as it may appear to a non‐expert. As such, our findings indicate that some Internet sources, such as online news, are likely to perpetuate negative stereotypes or attitudes about genetically engineered foods. The precise effect of these varied portrayals of GE food on public opinion remains to be determined.

Framing of agricultural biotechnology in mass media changes over time.^11^ Therefore, periodic reviews of GE food‐related information online, such as that described here, can help expert communities understand how contemporary opinions about GE food are influenced. This information can assist efforts by the scientific community to promote the prudent use of agricultural biotechnologies. For example, the empirical evidence described in this study can be incorporated into public discussions about GE food knowledge, attitudes and perceptions.

Furthermore, the rapid development of digital technology allows the public to use social media to express their opinions. The public discourse emerging from social media can be regarded as a reflection of the ideas disseminating from mass media. In the future, analysis of the coevolution of social discourse on Web‐based public spaces and the content of mass media can provide a more accurate way to measure how contemporary opinions about GE food are influenced.

## Conflict of Interest

The authors declare no conflict of interest.
